# Mitochondrial-Derived Peptides in Diabetes and Its Complications

**DOI:** 10.3389/fendo.2021.808120

**Published:** 2022-02-03

**Authors:** Ying Wu, Liankun Sun, Zhoudao Zhuang, Xiaoqing Hu, Delu Dong

**Affiliations:** ^1^ Key Laboratory of Pathobiology, Ministry of Education, Department of Pathophysiology, College of Basic Medical Sciences, Jilin University, Changchun, China; ^2^ Clinical Medical College of Jilin University, The First Hospital of Jilin University, Changchun, China; ^3^ Department of Ophthalmology, The First Hospital of Jilin University, Changchun, China

**Keywords:** mitochondrial-derived peptides (MDPs), humanin, MOTS-c, SHLPs(1-6), stroke, myocardial infarction, diabetes

## Abstract

The changes of mitochondrial function are closely related to diabetes and its complications. Here we describe the effects of mitochondrial-derived peptides (MDPs), short peptides formed by transcription and translation of the open reading frame site in human mitochondrial DNA (mtDNA), on diabetes and its complications. We mainly focus on MDPs that have been discovered so far, such as Humanin (HN), mitochondrial open reading frame of the 12S rRNA-c (MOTS-c) and Small humanin-like peptides (SHLP 1-6), and elucidated the role of MDPs in diabetes and its major complications stroke and myocardial infarction by improving insulin resistance, inhibiting inflammatory response and anti-apoptosis. It provides more possibilities for the clinical application of mitochondrial derived peptides.

## Introduction

Mitochondria, as the integration center of key signals regulating bioenergy metabolism and regulating the initiation and execution of oxidative balance protein apoptosis, can sense cellular stress and help cells adapt to the challenges of microenvironment (1, 2). Mitochondria are important organelles involved in glucose metabolism and the main source of ROS in cells, and their functional changes are closely related to blood glucose level, which can cause oxidative stress when hyperglycemia occurs due to excessive production of peroxide in mitochondrial electron transport chain (3). Oxidative stress is widely believed to play a key mediating role in the development and progression of diabetes and its complications due to the increased production of free radicals and impaired antioxidant defense ability (4).

As a one of the world’s fastest growing disease, diabetes and its complications is a major cause of death in diabetes. The body long carbohydrate metabolism disorder can cause multiple system damage, lead to eyes, kidneys, nerves, heart, blood vessels and other tissues and organs of chronic progressive lesions. Common complications of diabetes mainly include Cardiovascular complications, Diabetic nephropathy, Diabetic foot, Diabetic retinopathy, etc. (5–7) among which cardiovascular diseases and neurological diseases (8, 9) are the main causes of disability and death in diabetic patients. Mitochondria are related to the occurrence and development of diabetes and its complications (10, 11). Hyperglycemia can cause increased generation of mitochondrial ROS (12), and then affect diabetic complications such as ischemic stroke (8) myocardial infarction. In addition to the above mentioned regulation of diabetic hyperglycemia by affecting glucose metabolism, current studies have found that open reading frame sites contained in human mitochondrial rRNA can encode and form polypeptides called mitochondrial-derived peptides (MDPs). MDPs (13, 14) can be used as a new type of reverse signal molecule, the cell will retrograde pass the signals to the nucleus during stress, the regulation of gene transcription synthesis, thereby exert anti-inflammatory antiapoptotic and promote the synthesis of mitochondrial biological effect and so on, which affect the development of diabetes and its complications. We mainly discuss MDPs and the correlation of diabetes, we found a retrospect of the polypeptide function and their relationship with diabetes mellitus and related complications, especially the two more studied HN and MOTS-c.

## A Mitochondrial-Derived Peptide Types and Functions

### Humanin

**Figure 1:**
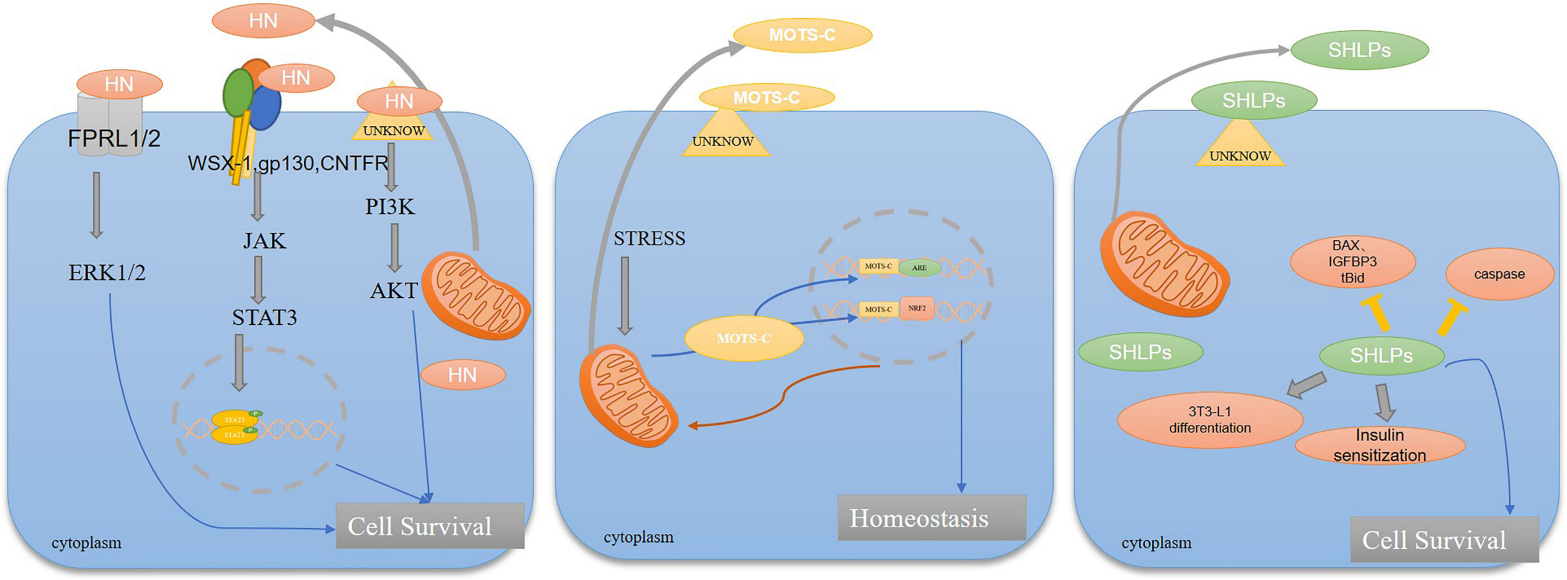
Role of mitochondria derived peptides in cells and their regulation of related diseases. HN acts by activating formylpeptide-like receptors (FPRL) and heterotrimeranthroinin receptors composed of GP130 ciliated neurotrophic factor receptor (CNTFR) and WSX-1. In addition, HN promotes phosphorylated STAT3 dimer entry into the nucleus to regulate gene transcription and activates the FPRL1/2-ERK1/2 pathway to play a protective role in cell. MOTS-c translocates to the nucleus in a 5’ -adenosine monophosphat-activated protein kinase (AMPK) -dependent manner after metabolic stress. MOTS-c regulates a wide range of genes in response to glucose restriction, including genes with antioxidant response elements (ARE), and interacts with stress response transcription factors that regulate ARE, such as the nuclear factor erythrocyte 2-associated factor 2 (NFE2L2/NRF2), to enhance mitochondrial function and thus maintain cellular homeostasis. SHLPs can enhance insulin sensitivity, promote adipocyte 3T3-L1 differentiation, inhibit the expression of caspase and ROS, and play a cellular protective role.

Humanin was first isolated and discovered by Japanese researcher Hashimoto (15) in the context of the protective factor of Alzheimer’s disease. It is composed of 24 amino acids encoded in the 16S rRNA region of mtDNA and transcribed by the mitochondrial multi-cistron gene MT-RNR2. Replacing Ser at position 14 with Gly produces a potent form of HN-derived S14G-humanin (HNG), which is more than 1000 times more active than naturally sourced HN (16). The mRNA of HN peptide contains 21 amino acids for mitochondrial translation and 24 amino acids for cytoplasmic translation (17), both of which have similar biological functions and share the same essential functional domains in HN secretion and cell protection. HN exists not only in circulating body fluids, such as blood and cerebrospinal fluid, but also in metabolically active organs and tissues, such as heart, liver, and kidney, as well as neurons and skeletal muscles (16, 18, 19). The HN has three regions, including the negatively charged C-terminal (PVKRRA), the positively charged N-terminal (MAPR), and a central hydrophobic region (GFSCLLLLTSEIDL) (20) that can bind hydrophobic pockets of proteins to form alpha helix (21). HN acts by activating formyl peptide-like receptors such as (FPRL) (22) and heterotrimeric humanin receptor (23, 24) composed of gp130, ciliary neurotrophic factor receptors (CNTFR), and WSX-1. HN binding to extracellular formylpeptide receptor-like 1/2 (FPRL1/2) induces increased Ca^2+^ flux and cascading activation of extracellular signal-regulated kinases (ERK 1/2) and downstream signals, resulting in anti-apoptotic effects, and thus improved cell survival. HN binds to Gp130 WSX-1 and CNTFR receptors, and trimerization of the receptors induces activation of Janus kinases (JAK1 and JAK2), which in turn activate signal transduction factors and transcriptional activator 3 (STAT3). Dimerized STATS translocations to the nucleus to regulate target gene transcription and play a protective role in cells (25). HN also co acts with insulin-like growth factor binding protein 3 (IGFBP-3) (26) to inhibit IGFBP-3-induced apoptosis (27). Currently, HN is mainly derived from exogenous sources. In retinal pigment epithelium (RPE) cells, the exogenous HN is located in mitochondria and can promote the secretion of endogenous HN (28). The current study has found that HN, as a retroactive signal peptide molecule produced by mitochondria, has certain anti-inflammatory (29), and anti-apoptotic (30, 31), effects, promotes mitochondrial biosynthesis, enhances signal molecules in insulin-mediated Akt signaling pathway and fatty acid metabolism signaling pathway, and regulates metabolism related to aging (**Figure 1**).

### MOTS-c

MOTS-c sequences, which were discovered by Lee J et al. (14), are highly conserved, especially for the first 11 residues, and are the first clear example of a reverse signal peptide molecule that can enter the nucleus and affect the stress transcriptional response. Studies have found that MOTS-c, as a reverse signal transgenic molecule-regulating gene transcription, translocates into the nucleus and binds to DNA under stress, and works with other transcription factors such as ARE to regulate the transcription of stress response genes, enhance cellular resistance and maintain homeostasis *in vivo* (32–35). MOTS-c is mainly extranuclear and co-localized in mitochondria under resting conditions (14, 32). However, during metabolic or oxidative stress, MOTS-c can be rapidly translocated to the nucleus in an AMPK-dependent manner within 30 min (32). MOTS-c entry into the nucleus requires a hydrophobic core, and in the nucleus, MOTS-c is able to bind chromatin through its hydrophobic and positive regions, as well as adaptive stress response transcription factors, including NFE2L2,Nrf2 and activating transcription factor 1 (ATF1) (32). MOTS-c is expressed in various organs and tissues of rodents and human skeletal muscle, myocardial kidney, and circulating plasma. MOTS-c has been shown to inhibit *de novo* purine synthesis, activate AMPK, and regulate fatty acid metabolism *in vivo* (14). MOTS-c also prevents coronary endothelial dysfunction by inhibiting NF-κB and reducing the release of pro-inflammatory cytokines and adhesion molecules. MOTS-c also has a regulatory effect on aging, insulin resistance caused by glucose metabolism disorders, and other aspects (**Figure 1**).

### SHLP (1-6)

SHLPs are 20-38 amino acid long peptide sequences encoded by mitochondrial 16S rRNA, which are divided into six types, SHLP1-SHLP6.Of these SHLP2 and SHLP3 are widely studied and have similar protective effects to Humanin. SHLP2 is mainly found in liver. The expression of SHLP3 is high in the kidneys and muscles, while SHLP3 is mainly high in the brain and spleen (36). Studies have shown that SHLP2 can increase the signal of the insulin-mediated Akt pathway and fatty acid metabolism signaling pathway, thus maintaining the homeostasis of glucose metabolism and fatty acid metabolism. SHLP2 can also increase the number of pancreatic cells, improve mitochondrial bioenergy, and participate in a chaperon-like effect (36, 37). It also reduces apoptosis by downregulating the effect of caspase family on age-related macular degeneration cells (31). With the increase in age, SHLP2 level in blood circulation gradually decreases, suggesting that it is related to the progression of age-related diseases (36). SHLP3 can inhibit ROS production, mediate ERK signal transduction, and promote adipose cell differentiation. In addition, SHLP2/SHLP3 can play an insulin sensitization role *in vitro*, enhance the ability of insulin to inhibit glucose production in the liver, promote the peripheral disposal of glucose, and play a role in regulating glucose metabolism homeostasis. SHLP2 and SHLP3 can also enhance cell viability and reduce cell apoptosis, while SHLP6 can do the opposite (36). In addition, SHLP2 and SHLP3 can block mitochondrial membrane damage induced by staurosporine (STS) and activation of caspase-3, thus playing a protective role (38) (**Figure 1**).

## Mitochondrial-Derived Peptides and Disease

### Mitochondrial-Derived Peptides and Diabetes

Diabetes mellitus (DM) (39, 40) is mainly divided into type 1 diabetes mellitus with absolute insulin deficiency caused by the destruction of pancreatic beta cells, type 2 diabetes mellitus with insulin resistance (41), and other special types of diabetes mellitus, according to clinical manifestations, pathophysiology, and etiology. Current treatments for diabetes include hyperglycemic drugs such as oral hypoglycemic agents, insulin, exercise therapy, and surgery. In metabolic tissues with insulin resistance (42), abnormal mitochondrial morphology is often found, the number of mitochondria and their oxidase is reduced, and the production of ATP is reduced. The accumulation of high circulating free fatty acids in these tissues also reduces glucose processing in response to insulin stimulation.

Present studies have demonstrated that, by targeting skeletal muscle, MDPs have a mitigating effect on insulin resistance and induce glucose uptake into the pentose phosphate pathway to avoid hepatotoxicity caused by drugs such as metformin (43) or methotrexate. Among them, Humanin has the ability to bind insulin-like growth factor binding protein 3 (26). Humanin’s entry into the ventricle leads to increased insulin sensitivity in the liver and muscle, resulting in reduced glucose production in the liver and increased insulin mediated Akt signaling and fatty acid metabolism signaling. Humanin also enhances peripheral glucose uptake and inhibits liver glucose production (44, 45). Han et al. showed that HNG may improve insulin resistance by reducing Ser636 phosphorylation of insulin receptor substrate 1 (IRS1) in the hippocampus. In addition, SHLP2 and SHLP3 can also improve insulin response, enhance the ability of insulin to inhibit glucose production in the liver, and promote peripheral glucose processing. In 2016, Cobb LJ et al. (36) found the insulin sensitization effect of SHLP2 and SHLP3 *in vitro* and *in vivo*. In response to insulin, SHLP2 and SHLP3 both accelerated the differentiation of 3T3-L1 cells in mouse pre-adipose cell lines and enhanced insulin sensitivity. Compared with SHLP3, SHLP2 improved insulin responsiveness, enhanced insulin’s ability to inhibit hepatic glucose generation (HGP), and promoted glucose access to peripheral tissues. MOTS-c targets skeletal muscle, and thus can enhance systemic insulin sensitivity, improve glucose processing rate, and promote AMPK activation and GLUT4 expression through muscle. In 2015, Lee et al. found that MOTS-c can promote AMPK activation and GLUT4 expression under high-fat diet (HFD), enhance systemic insulin sensitivity through muscle, and increase the glucose processing rate of insulin stimulation (14). Lu et al. demonstrated for the first time that MOTS-c treatment can prevent ovariectomy-induced insulin resistance, fat deposition and inflammatory response in mice (46). After oophorectomy, estrogen deficiency increases fat load and disrupts normal fat function, thus forcing insulin resistance. MOTS-c regulates fat metabolism by increasing energy consumption and inhibiting fat bulge, thus alleviating diabetes caused by insulin resistance. In addition, Zhai et al. observed that in mice infected with methicillin-resistant Staphylococcus aureus (MRSA), MOTS-c enhanced phagocytosis and bactericidal capacity of macrophages by inhibiting MAPK, enhancing expression of negative regulator of inflammation AHR and phosphorylation of STAT3. At the same time, the levels of pro-inflammatory cytokines TNF-α, IL-6, and IL-1β decreased, and the levels of anti-inflammatory cytokine IL-10 increased (47, 48). Thus, MDPs provides a new direction for the treatment of insulin resistance associated with inflammation.

### Mitochondrial-Derived Peptides and Stroke

Stroke is a major complication of diabetes. As an acute cerebrovascular disease, it is a group of diseases caused by brain tissue damage due to sudden rupture of blood vessels in the brain or inability of blood flow to the brain due to vascular obstruction, including ischemic and hemorrhagic stroke (49). In ischemic stroke, tight junction proteins of vascular endothelial cells are degraded, and changes in BBB permeability lead to the activation of immune cells, which then penetrate into endothelial cells and infiltrate brain tissue, thereby triggering an inflammatory cascade that leads to neuronal damage and cell death. Recent studies have confirmed that HN (50) plays a protective role against ischemic brain injury. HN can pass the blood-brain barrier (BBB) and regulate NF-κB (25) PI3K-Akt, JAK-Stat3, and other pathways (49), or regulate the expression of apoptotic related proteins to inhibit neuronal apoptosis, thus playing a protective role against ischemic brain injury. Peng et al. (49) conducted *in vivo* experiments on mice with *in vivo* middle cerebral artery occlusion (MACO) model and *in vitro* studies on Bend3 cells treated with hypoxia and glucose deficiency, showing that HNG, a reverse signaling molecule, can reduce inflammatory response *in vivo* by inhibiting the activation of NF-κB signaling pathway factors IKK, and IKB, and reducing the accumulation of P65 in the nucleus. For example, HN can inhibit the production of cytokines such as tumor necrosis factor α (TNF-α) and interleukin 1β (IL-1β), while at the same time inhibiting vascular adhesion molecules in cortical tissues such as VCAM-1 and ICAM-1. It was speculated that the disorder of BBB endothelial cells in the brain of MCAO mice might promote the passage of HNG through BBB. Moreover, in a 2016 study, Kim et al. (23) found that in neuronal cell lines, HNG can activate Akt Erk1/2 and Stat3 signaling pathways through the glycoprotein 130kDa (GP130/IL6ST) receptor complex to play a protective role in nerve cells. HNG can inhibit oxidative stress ROS production by activating the JAK2/STAT3 signal and the mitochondrial pathway-related apoptosis induced by Bax and caspase3 (25). HN inhibits BAX-mediated neuronal apoptosis mainly through two pathways (51). First, HN prevents the translocation of Bax from cytoplasm to mitochondria. Second, HN interacts with the mitochondrial membrane bound to Bax to prevent the recruitment of cytoplasmic Bax and its oligomerization in the membrane.

### Mitochondrial-Derived Peptides and Myocardial Infarction

Another major complication of diabetes, myocardial infarction (52), refers to acute myocardial ischemic necrosis, mostly on the basis of coronary artery lesions, the coronary artery blood supply is sharply reduced or interrupted, resulting in severe and lasting acute ischemia of the corresponding myocardium. Current treatments include drug therapy, interventional medicine and surgery. Nevertheless, the traditional method of treatment has been unable to meet the needs of clinical patients. Therefore, it is necessary to seek efficient and reliable treatment. Current studies have found that as an important peptide for regulating and maintaining mitochondrial function, MDPs (53) can be involved in the pathological changes in cardiovascular disease (CVD) through different mechanisms. The heart is an organ with high internal oxygen consumption, and ROS are mainly produced by cardiac mitochondria. To be specific, the complex of the electron transport chain (ETC) is the main source of ROS produced by cardiac mitochondria (54). ROS are multipotent, and in relatively high concentrations (pathology) cause oxidative stress, but at a lower level (physical) act as a signal molecules. The increase of ROS will lead to changes in mitochondrial membrane potential and ATP level of cardiomyocytes. At the same time, oxidative stress can trigger mitochondria and endoplasmic reticulum stress mediated apoptosis pathways, causing cell damage.HN can protect cells and mitochondria through antioxidant stress and endoplasmic reticulum stress. Savitree and his colleagues demonstrated that HNG can reduce mitochondrial damage caused by complex I and reduce oxidative stress caused by H_2_O_2_ and ROS production (55). Moreover, HNG was more effective than cyclosporine A (CsA, MPTP inhibitor) in reducing mitochondrial ROS and increasing ATP production. It has been found in a series of studies (56) that high dose HNG (252 ug/kg) can increase the HN level of damaged myocardium and reduce arrhythmias, area of myocardial injury and mitochondrial dysfunction. In addition, Laura E. Klein et al. (57) also found that the reduction of intracellular ROS after HNG-treatment was dependent on the activation of a pair of non-receptor tyrosine kinases C-ABL and arginine. Their results provide mechanistic insights into the observed HNG-mediated cardiac protection *in vivo* (58). Glutathione (GSH) is an important component of mitochondrial antioxidant defense system. Matsunaga et al. (59) showed that HN can restore mitochondrial GSH synthesis by increasing the catalytic subunit of rate-limiting glutamylcysteine ligase and inhibiting the production of superoxide, thus protecting against various ER stress-induced apoptosis. In addition, Muzumdar RH et al. (60) found that HNG may activate AMPK-eNOS -mediated (endothelial nitric oxide synthase) signal transduction during myocardial ischemia-reperfusion injury (MI-R model) in mice. Activating AMPK, increasing the phosphorylation level of eNOS, and decreasing the expression of the apoptotic factor Bax can help reduce the myocardial infarction area, enhance cardiac function, improve the survival rate of myocardial cells, and play a cardiac protective role in a dose-dependent manner (HNG,2 mg/kg is the best) in the MI-R mouse model. Yuan et al. (61) investigated the effects of MOTS-c on cardiac function and structure of rats during chronic aerobic exercise by intraperitoneal injection of MOTS-C, and detected echocardiography by HEMO dynamics with HE staining. After analyzing cardiac function, it was found that MOTS-c could improve cardiac mechanical efficiency, enhance cardiac systolic function, and have a tendency to improve diastolic function, thus improving cardiac function.

## Conclusion

**Figure 2:**
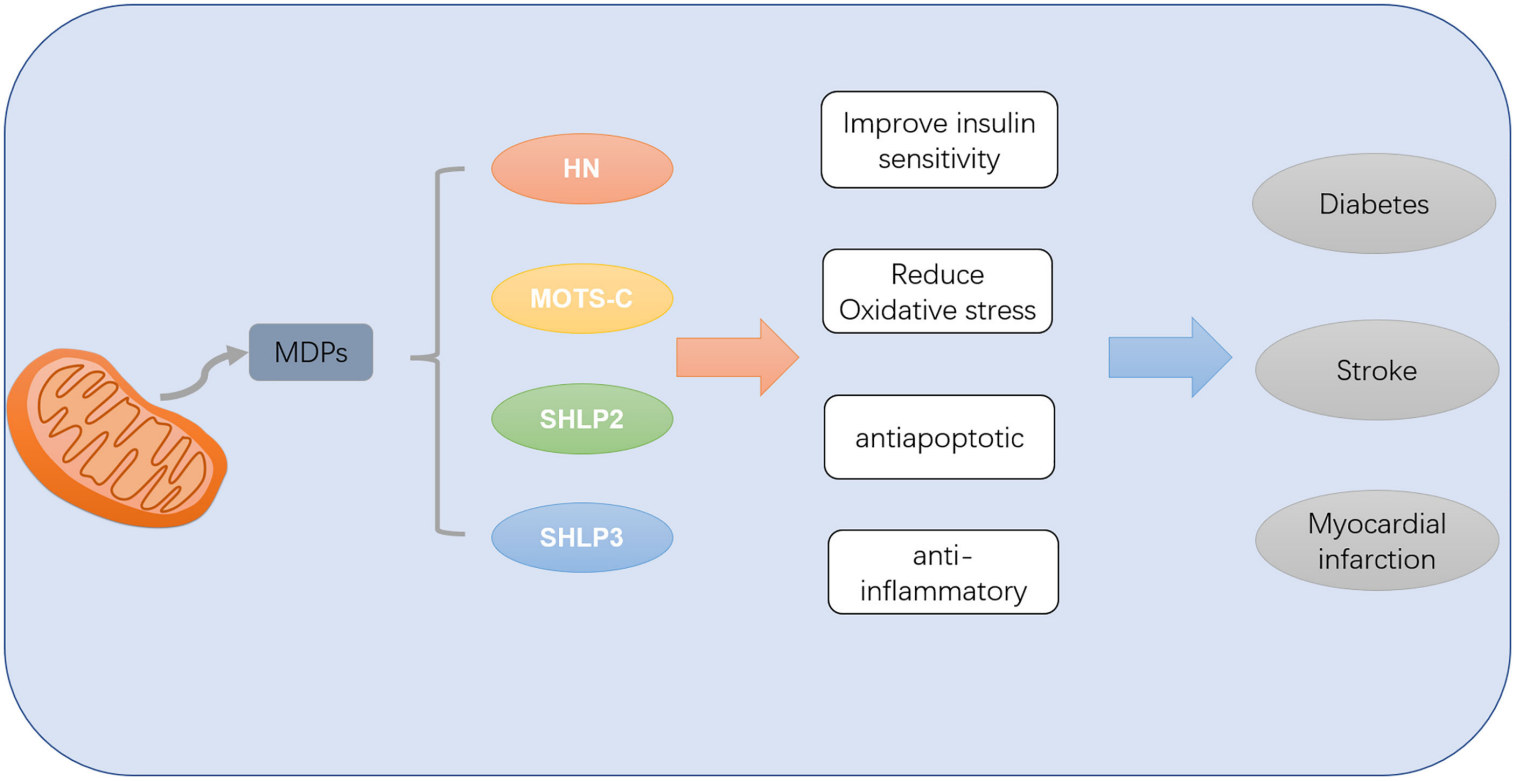
MDPs can improve diabetes mellitus and its complications of myocardial infarction and stroke by improving insulin sensitivity and reducing oxidative stress inflammation and apoptosis.

Mitochondria are the energy metabolism center in the body, while brain and heart are the most metabolically active organs in the body. Therefore, changes in mitochondrial function will affect the process of diabetes and its complications in the heart and brain tissues (62). ROS produced by mitochondria in the process of metabolism is considered to be the main cause of diabetic microangiopaxia caused by mitochondrial mutation damage to aging tissues. MDPs encoded by mitochondrial genes can regulate diabetes and its cardiovascular and cerebrovascular complications through anti-inflammatory and anti-apoptosis promotion of mitochondrial biosynthesis, etc. (**Figure 2**). HN (23) can reduce ROS interference with BAX translocation and recruitment through activation of PI3K-AKT, JAK2-STAT3, NF-κB, AMPK-eNOS and other pathways. MOTS-c (58) can regulate glucose and lipid metabolism by targeting specific activation of AMPK in skeletal muscle, regulate coronary endothelial function, enhance cardiac systolic function, improve coronary artery microvascular disorders, etc. SHLP2 and SHLP3 can also improve insulin sensitivity and glucose metabolism *in vivo* and *in vitro*, thus contributing to the efficacy of diabetes and its complications.

Mitochondrial derived peptides mainly play a role in the regulation of diabetic nervous system complications by HN, while other MDPs have not been fully reflected in the study of neuroprotection. In addition, most of the mitochondria derived peptides used in the current research are exogenous. As protein polypeptides, they can be quickly cleared by tissues in the body, so how to make them play a role in the body for a long time is also one of the problems that need to be solved.

## Author Contributions

Conceptualization, DD and XH. Original draft preparation, ZZ. Visualization, LS. Writing and editing, YW. All the authors read and approved the final manuscript.

## Funding

National Natural Science Foundation of China (81772794, 82102733, 82072206) Jilin Provincial Research Foundation for the Development of Science and Technology Projects (20200703009ZP, 20190201164JC) Jilin Provincial Health Technology Innovation Project (2021JC034, 2020Q010) Jilin Province Department of Finance (2019SRCJ022).

## Conflict of Interest

The authors declare that the research was conducted in the absence of any commercial or financial relationships that could be construed as a potential conflict of interest.

## Publisher’s Note

All claims expressed in this article are solely those of the authors and do not necessarily represent those of their affiliated organizations, or those of the publisher, the editors and the reviewers. Any product that may be evaluated in this article, or claim that may be made by its manufacturer, is not guaranteed or endorsed by the publisher.
